# Rest Assured: The Association of Structural, Functional Support, and Loneliness With Subjective Sleep Health

**DOI:** 10.1111/jsr.70303

**Published:** 2026-02-09

**Authors:** Eva De Camargo, Stephanie Schindler, Andrea E. Zülke, Heide Glaesmer, Andreas Hinz, Christoph Engel, Kerstin Wirkner, Steffi G. Riedel‐Heller, Georg Schomerus, Christian Sander

**Affiliations:** ^1^ Department of Psychiatry, Medical Faculty University of Leipzig Leipzig Germany; ^2^ Department of Psychiatry and Psychotherapy University Hospital Leipzig Leipzig Germany; ^3^ Institute for Social Medicine, Occupational Health and Public Health University of Leipzig Leipzig Germany; ^4^ Department of Medical Psychology and Medical Sociology University of Leipzig Leipzig Germany; ^5^ Institute for Medical Informatics, Statistics and Epidemiology University of Leipzig Leipzig Germany; ^6^ LIFE‐ Leipzig Research Centre for Civilization Diseases University of Leipzig Leipzig Germany

**Keywords:** functional support, German cohort, loneliness, PSQI, sleep disparity, structural support

## Abstract

Sleep is increasingly understood as a socially embedded phenomenon. This study examined how structural and functional aspects of social support, as well as loneliness, relate to sleep health in a German sample of middle‐aged adults (*N* = 5388). Drawing on the socio‐ecological model of sleep health, we assessed the contributions of social support dimensions while accounting for age, sex, and socioeconomic status, as well as psychological covariates. The results of the binary logistic regression showed that functional support (ESSI), friend network size (LSNS6), and loneliness (CES‐D item 14) significantly (*p* < 0.001) predicted sleep health (PSQI), while family network size did not. The portion of explained variance was small (4%–5%). Results remained robust after adjusting for age, sex, and socioeconomic status, but no longer when including psychological covariates (GAD‐7, SWLS, CES‐D), in which case only the friend network size remained significant (*p* = 0.019). Women were significantly more affected by poor sleep health than men, and with higher socioeconomic status, fewer people reported suffering from poor sleep (all: *p* < 0.001). Additional subgroup analysis revealed higher age as a risk factor for worse sleep health in women only, while the friend network was only relevant in men. Our findings highlight the importance of distinguishing between structural and functional dimensions of social support in sleep health research and interventions, and suggest a potential sex‐by‐age interaction. Future research should promote equity by including diverse populations and longitudinally examine how social support, especially friend networks, affects sleep across genders, ages, and contexts.

## 1 Introduction

This study focused on the association of social support and loneliness with sleep health in a German sample of adults between 40 and 80 years old. Traditionally studied through physiological and behavioural lenses, newer conceptual frameworks increasingly emphasise the influence of social and environmental factors on sleep. Relying on the socio‐ecological model of sleep health, we investigated social determinants and sociodemographic covariates.

### 1.1 Social Determinants of Sleep Health

Sleep is a behaviour crucial for mental and physical health that is often shared between people, such as romantic partners, roommates, parents, and kids. Conventional sleep research, however, has focused on the sole individual, rarely accounting for the relational context in which sleep occurs. Considering the social dimension of sleep opens new pathways for understanding how social networks and close relationships might influence sleep health.

Simultaneously, promoting sleep health represents an often overlooked opportunity in public health with significant potential to impact key health consequences (e.g., cardiovascular disease, obesity, mental health conditions) (Hale et al. 2020; Appleton et al. 2022). The concept of sleep health comprises various dimensions (e.g., regularity, alertness, timing, efficiency, and satisfaction) rather than focusing solely on isolated symptoms or disorders (Buysse 2014).

The socio‐ecological model of sleep health provides a framework for understanding how individual, interpersonal, socio‐demographic, and broader contextual factors interact to provide sufficiently adequate conditions for ‘good’ sleep (Hale et al. 2020). According to this model, sleep health is influenced at different levels, ranging from personal behaviour to interpersonal, community, and societal factors. At the individual level, healthy sleep behaviour − such as maintaining a consistent sleep schedule (Espie 2022; Beranek et al. 2025), limiting caffeine (Clark and Landolt 2017; Reichert 2022) and alcohol consumption (Helaakoski et al. 2022), as well as conscious phone use prior to bedtime (Eeftens et al. 2023) – can promote better quality of sleep. Additionally, socio‐demographic factors (e.g., race, ethnicity, socio‐economic background) play a role in sleep outcomes (e.g., Grandner et al. 2016). At a community level, environmental factors, such as noise (e.g., Smith et al. 2022), but also employment situation (e.g., Greissl et al. 2022), can impact sleep quality and patterns. Finally, the local, state, and national policies related to social welfare and healthcare (e.g., financial stress, safety, residential segregation) can possibly impact sleep health on the societal level (Hale and Hale 2009). This study positions itself at the interpersonal level of the socio‐ecological model.

### 1.2 Linking Social Support and Sleep Health

We define social support in accordance with the broad social support literature (Cobb 1976) as the perception of being cared for, valued, and part of a supportive network, emphasising the meaning individuals attribute to their network. Social support is strongly linked to key mental health outcomes, including depression and anxiety (Uchino et al. 2016), and has been linked to improved sleep outcomes (de Grey et al. 2018).

However, to our knowledge, the role of social support in relation to sleep health has only recently gained increased attention in research. As a consequence, there remains a notable gap in the literature regarding how social support influences sleep health specifically. While this area is still emerging, we assume that similar mechanisms apply as those established in the broader health literature, where social support is known to buffer stress, promote emotional regulation, and enhance overall well‐being, the so‐called stress‐buffering and direct effect models (Cohen and Wills 1985; Uchino 2004): Stress‐related models, based on Lazarus and Folkman (1984), suggest that support helps by reducing perceived stress or weakening its impact on mental health. The direct effect model, on the other hand, proposes that social support promotes mental well‐being more broadly by fostering connection, esteem, and a sense of control, regardless of stress levels. Both pathways likely contribute to the relation between support and sleep health, though through distinct mechanisms. One study exploring the direction of the support‐sleep link suggests that receiving more support enhances satisfaction, which strengthens meaning in life, reduces depressive symptoms, and increases the likelihood of getting adequate sleep (Krause and Rainville 2019).

### 1.3 Social Support and Sleep Health

As a multidimensional construct (Cohen and Wills 1985), social support can be measured as *structural support* (e.g., type, amount, and degree of network ties) and *functional support* (e.g., quality of emotional support provided by networks). In addition, Barrera's (1986) influential review highlighted the distinction between *perceived* and *received* functional support. To provide conceptual clarity, we distinguish between structural and functional forms of support. **Structural support** refers to the quantitative aspects of an individual's social network, while **functional support** encompasses the *received* qualitative dimension (emotional closeness, and companionship). **Loneliness**, in addition, is defined in line with van Baarsen et al. (2001) as the *perceived* discrepancy between desired and actual social connections − a subjective experience of lacking meaningful ties, regardless of objective social contact. This distinction is crucial, as individuals with large networks may still feel lonely or deprived of meaningful social connections, whereas those with fewer contacts may not, depending on their expectations and needs.

The detrimental impact on sleep of both reduced social support and loneliness is supported by a small body of previous research on older adults, suggesting that loneliness is linked to poorer sleep quality (Azizi‐Zeinalhajlou et al. 2022; Gyasi et al. 2022; Deng et al. 2023), social support (cohabiting with another person) to enhanced sleep quality (McLay et al. 2022), and high perceived support to improved sleep quality (Nomura et al. 2010; Troxel et al. 2007).

Our study aims to examine associations between loneliness, structural support (network size, further distinguishing between family and friend networks), and functional support with sleep health. We assume that the more acquaintances to rely on (i.e., higher structural support) and the better the felt connection in this network with each member (i.e., higher functional support), the better the sleep health outcome. In addition, we conducted a stratified analysis by sex and socioeconomic status (SES), hypothesising to find sex differences and differences between high and low SES groups: The positive effect of the social network on sleep health would be higher in more stressful life conditions such as being a member of a low‐income group or having female sex (Åkerstedt et al. 2016).

## 2 Methods

We used a cross‐sectional design to examine in how far social support and loneliness are related to sleep health in a large, community‐based adult sample in Germany. Drawing on the socio‐ecological model of sleep health, we focused on interpersonal‐level predictors as key variables of interest. The analyses accounted for demographic and psychological covariates. Additional subgroup analyses explored effects stratified by sex and SES. The current analyses were not preregistered. Details on the data, operationalization of variables, and statistical approach are outlined below.

### 2.1 Data

Data stemmed from the large‐scale LIFE‐Adult‐Study (Leipzig Research Centre for Civilization Diseases), which recruited a population‐based sample of 10,000 participants (18 to 80 years) between August 2011 and November 2014 in Leipzig, Germany (Loeffler et al. 2015). During the visit to the LIFE study centre, information on sociodemographic factors, medical history, and subjective well‐being was collected using questionnaires and standardised interviews performed by trained raters. Information on medical history was obtained by asking about the occurrence of specific diseases. Alcohol consumption was assessed by inquiring about the amount and frequency of consumption within the last year. The medication taken was documented by recording the ATC codes of the medication currently in use. All participants gave written informed consent to participate in the study. The procedures were conducted according to the Declaration of Helsinki and approved by the University of Leipzig Medical Faculty's ethics committee (registration number: 263‐2009‐14122009).

#### 2.1.1 Inclusion Criteria

**FIGURE 1:**
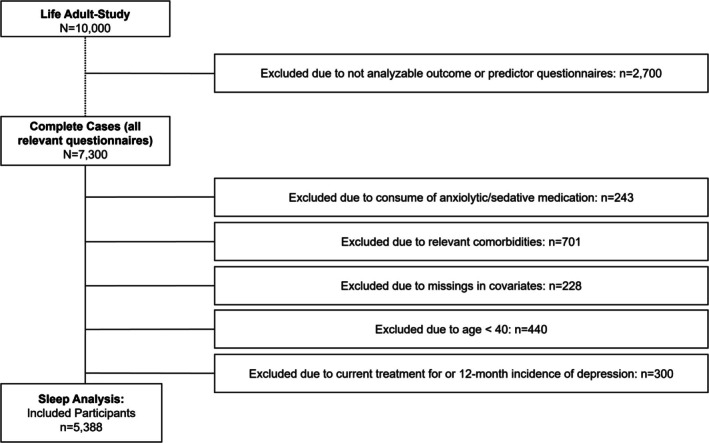
Flow *chart for inclusion of participants into the analyses*. The figure demonstrates the inclusion process of participants in the analyses. Complete cases = Participants who had completed the questionnaires for the outcome and predictors. Outcome = PSQI Score; Predictors = ESSI Score, LSNS‐6 Score, Item 14 of CES‐D; Anxiolytic/sedative medication = anaesthetics, opioids, anxiolytics, benzodiazepines, hypnotics, sedatives; Relevant comorbidities = current treatment of gout, rheumatic diseases, Crohn's disease; lifetime diagnosis of Parkinson's disease, epilepsy, multiple sclerosis, Crohn's disease, dialysis dependency; 12‐month incidence of stroke, tuberculosis, cancer, hepatitis, myocardial infarction; Covariates = SWLS, GAD‐7, CES‐D, sex, age, and SES.

Figure 1 summarises the inclusion procedure along with relevant details on the exclusion criteria applied. From the LIFE data set, we selected only those participants who had completed the sleep health as well as all three predictor questionnaires, as calculation of the total scores required complete responses. Missingness was assumed to be due to examinations not performed or accidentally skipped items. Therefore, apart from depressive symptoms (see Section 2.2), missing values in neither outcome nor predictors were imputed. Participants consuming anxiolytic or sedative medication were excluded, as were participants with severe comorbidity impacting sleep − for example, current treatment for gout or rheumatic diseases, a lifetime diagnosis of Parkinson's disease or epilepsy, or a 12‐month incidence of stroke or cancer. Due to their low representation in the sample, participants under the age of 40 were excluded. Finally, participants currently in treatment for depression were excluded in an attempt to limit their well‐established confounding effect on sleep.

#### 2.1.2 Outcome: Sleep Health

Participants were asked to evaluate their sleep health using the Pittsburgh Sleep Quality Index (PSQI; Buysse et al. 1989). The PSQI score includes the subscales sleep quality, latency, duration, efficiency, disturbances, use of sleep medication, and daytime dysfunction. Thus, the questionnaire serves as a global measure of sleep health (Buysse 2014). A global PSQI score (range = 0–21) over 5 indicates poor sleep health, whilst a score over 10 suggests a clinically relevant sleep disturbance. For the logistic regression analyses, sleep health was dichotomised into two categories (*good* vs. *poor*) using the cut‐off of 5, as we were interested in detecting social determinants of unhealthy, poor sleep and not merely clinically relevant sleep disturbances.

#### 2.1.3 Predictors: Social Support Variables

Structural support was assessed by asking for social network size using the brief version of the Lubben Social Network Scale (LSNS‐6; Lubben et al. 2006). The LSNS‐6 total score (range = 0–30) assesses different aspects of social interaction: higher scores reflect a larger social network, while scores of 12 or lower are considered indicative of risk for social isolation. Participants were asked to assess their functional support by using the Enriched Social Support Instrument (ESSI; Berkman et al. 2003). Higher values in the sum score (range = 5–25) indicate more social support, while scores lower than 19 indicate insufficient social support. Loneliness was assessed using item 14 of the Center for Epidemiologic Studies Depression Scale (CES‐D), which asks about feelings of loneliness during the past week. Responses range from 0 (‘rarely or none of the time’) to 4 (‘most or all of the time’).

#### 2.1.4 Covariates

Sex (binary), age, SES, anxiety symptoms, and life satisfaction were added as covariates to test the robustness of effects. SES was calculated as a multidimensional index based on education (school, professional), occupational status, and equivalent household income in line with the procedure suggested by Lampert et al. (2013). According to their recommendation, SES subgroups were defined as follows: low = 3–9.2 points, middle = 9.3–15.3 points, high = 15.4–21 points. Anxiety symptoms over the past 2 weeks (range = 0–21) were assessed using the Generalised Anxiety Disorder Screener (GAD‐7; Loewe et al. 2008), with sum scores from 0 to 4 indicating minimal, 5 to 9 mild, 10 to 14 moderate, and 15 or severe anxiety. Life satisfaction was assessed using the 7‐item Likert Satisfaction with Life Scale (SWLS; Diener et al. 1985): lower sum scores (range = 5–35) indicate less life satisfaction. In line with the above‐mentioned criteria, in a German population sample, scores < 19 represented a below‐average life satisfaction (Glaesmer et al. 2011). Depressive symptoms were assessed using the CES‐D (Radloff 1977), a self‐report scale measuring symptom severity over the past week.

### 2.2 Statistical Analysis

A hierarchical logistic regression analysis was conducted to examine the predictive value of social support and loneliness for poor sleep health (binary PSQI; 1 = poor sleep health). In the first model, all relevant social support variables were included. Loneliness was treated as a categorical predictor, with *never lonely* (0) serving as the reference category. In the second model, demographic covariates − sex, SES, and age − and in a third model, psychological variables − GAD‐7 score, SWLS score, and CES‐D score − were added. For comparability between predictors in the logistic regression models, we rescaled the SWLS score to a range of 0–30 and those of the ESSI score to a range of 0–20 (both by subtraction of 5 points). CES‐D scores were calculated excluding item 14 (loneliness) and item 11 (sleep disturbances) to avoid overlap with predictors and the outcome. In case of only one missing item, this was imputed using the mean imputation.

In the descriptive analysis, we report the original score values of both CES‐D and ESSI to allow for interpretation based on established norms. SES was inserted in models 2 and 3 as a metric variable. Lampert et al. (2013) designed the SES variable both as a metric and a categorical variable (with 3 subgroups). We used the latter for a more intuitive interpretation during subgroup analyses, which were conducted separately for sex and SES groups to explore potential differences.

In addition, we provide results of the omnibus Wald test for the categorical predictor loneliness, if needed. We also calculated predicted differences (average marginal effects) per coefficient and model. These can be interpreted as the percentage of more or fewer people reporting poor sleep per one‐point increase in the respective predictor. We checked the model for collinearity and inspected the standardised residuals. Correlations between the predictor variables in the multiple logistic regression analysis were all *r* < ±0.40 in size, except for the correlations between depressive symptoms with anxiety (*r* = 0.57) and with life satisfaction (*r* = −0.42). Cronbach's Alphas for the predictors and outcome were good (all > 0.72). Data were preprocessed and analysed by Statistical Package for the Social Sciences (SPSS) software (IBM SPSS Statistics 29.0.0.0). Additionally, average marginal effects and omnibus Wald tests were calculated using Stata 17.

## 3 Results

### 3.1 Sample Characteristics

A total of 5388 healthy adults from the LIFE sample (52.5% female, average age of 55.3 years) were analysed, with women being descriptively 2 years younger than men (Table 1). The largest age group was 40 to 49 years old (37.3%), while the smallest represented those aged 70 years or older (11.9%). The majority of the sample was married (65.0%), and most lived with a partner (78.0%). The most common SES group was the moderate SES category (61.6%), with women being descriptively underrepresented in the high SES category (22.5%) compared to men (27.5%). Overall sleep health was reported as *good* by 65.2% of the sample, but this rate was lower among women, with only 59.8% reporting good sleep (as opposed to 71.1% in men).

**TABLE 1:** Sample description, separated by sex.

	Total sample	Men	Women
*N* (%)	5388	2558 (47.5)	2830 (52.5)
Age, *M* (SD)	55.33 (10.3)	56.00 (10.6)	54.72 (10.0)
Age group, *n* (%)			
40–49 years	2012 (37.3)	916 (35.8)	1096 (38.7)
50–59 years	1453 (27.0)	666 (26.0)	787 (27.8)
60–69 years	1283 (23.8)	614 (24.0)	669 (23.6)
70+ years	640 (11.9)	362 (14.2)	278 (9.8)
Marital status, *n* (%)			
Married	3503 (65.0)	1713 (67.0)	1790 (63.3)
Single	894 (16.6)	478 (18.7)	416 (14.7)
Divorced	761 (14.1)	319 (12.5)	442 (15.6)
Widowed	230 (4.3)	48 (1.9)	182 (6.4)
Living situation, *n* (%)			
Living alone	1188 (22.0)	461 (18.0)	727 (25.7)
Living with a partner	4200 (78.0)	2097 (82.0)	2103 (74.3)
SES, *n* (%)			
Low	729 (13.5)	343 (13.4)	386 (13.6)
Moderate	3318 (61.6)	1512 (59.1)	1806 (63.8)
High	1341 (24.9)	703 (27.5)	638 (22.5)
Sleep Health (PSQI), *n* (%)			
Good	3511 (65.2)	1818 (71.1)	1693 (59.8)
Significantly bad	1548 (28.7)	651 (25.4)	897 (31.7)
Clinically relevant	329 (6.1)	89 (3.5)	240 (8.5)

*Note:* Description of the sample's demographic characteristics. Sleep health is presented here in its typical clinical three‐group classification, though a binary outcome was used in the statistical analyses.

Abbreviations: *PSQI* = Pittsburgh Sleep Quality Index, *SES* = socioeconomic status.

The loneliness levels were generally low across the whole sample (83.5% indicated being never lonely), while structural social support (*M* = 16.93, SD = 5.14) and functional support (*M* = 22.34, SD = 3.45) were relatively high. The life satisfaction of the sample was slightly above average (*M* = 26.96, SD = 5.14), especially when compared to reference scores (for the German version: *M* = 25), while anxiety (*M* = 3.10, SD = 2.9) was mild, and depressive symptoms (*M* = 9.54, SD = 6.0) were clinically within normal limits.

Descriptive analysis revealed that poor sleepers came from a slightly lower socioeconomic background (Table 2), were more often female (60.6%), and reported less often *never being lonely* (75.7%), had slightly smaller social networks, both in terms of family (*M* = 8.57, SD = 3.0) and friends (*M* = 7.50, SD = 3.3), reported slightly less functional support (*M* = 21.63, SD = 3.9), higher anxiety (*M* = 4.42, SD = 3.4), slightly lower life satisfaction (*M* = 25.30, SD = 5.7), and more depressive symptoms (*M* = 11.91, SD = 6.8) than good sleepers.

**TABLE 2:** Predictors of sleep health, separated by good and poor sleep health.

	Total sample	Good sleepers	Poor sleepers
*N*	5388	3511	1877
Sex (% female)	52.5	48.2	60.6
Age, *M* (SD)	55.33 (10.3)	55.15 (10.4)	55.66 (10.1)
SES, *M* (SD)	13.07 (3.3)	13.35 (3.2)	12.55 (3.2)
Never lonely (CESD‐14), *n* (%)	4498 (83.5)	3077 (87.6)	1421 (75.7)
Sometimes lonely (CESD‐14), *n* (%)	694 (12.9)	348 (9.9)	346 (18.4)
Often lonely (CESD‐14), *n* (%)	151 (2.8)	66 (1.9)	85 (4.5)
Mostly lonely (CESD‐14), *n* (%)	45 (0.8)	20 (0.6)	25 (1.3)
Struct. support (LSNS‐6), *M* (SD)	16.93 (5.1)	17.40 (5.0)	16.07 (5.3)
Family network, *M* (SD)	8.92 (2.9)	9.11 (2.8)	8.57 (3.0)
Friend network, *M* (SD)	8.01 (3.3)	8.29 (3.2)	7.50 (3.3)
Functional support (ESSI), *M* (SD)	22.34 (3.5)	22.72 (3.1)	21.64 (3.9)
Anxiety (GAD‐7), *M* (SD)	3.10 (2.9)	2.39 (2.4)	4.42 (3.4)
Life satisfaction (SWLS), *M* (SD)	26.96 (5.1)	27.84 (4.6)	25.30 (5.7)
Depressive symptoms (CES‐D), *M* (SD)	9.54 (6.0)	8.27 (5.0)	11.91 (6.8)

*Note:* Description of sample, key predictors, and covariates stratified by sleep health (good vs. poor sleepers).

Abbreviations: CES‐D = Center for Epidemiologic Studies Depression Scale; CES‐D‐14 = Item 14 of the Center for Epidemiologic Studies Depression Scale; ESSI = Enriched Social Support Instrument; GAD‐7 = Generalised Anxiety Disorder Screener; LSNS‐6 = Lubben Social Network scale; SES = socioeconomic status; SWLS = Satisfaction with Life Scale.

### 3.2 Results of Hierarchical Logistic Regression Model With Social Support Variables

Apart from family network size, all predictors were statistically significant (Table 3, model 1): As hypothesised, with each one‐point increase in functional social support, 1.1% fewer people and with each additional member of the friend network 1.1% fewer people reported poor sleep. Compared to individuals who reported *never feeling lonely*, 13.6% more people experienced poor sleep when they reported feeling *sometimes lonely*, 17.5% when feeling *often lonely*, and 14.4% when feeling *mostly lonely*. In addition, the omnibus test revealed a significant association of loneliness with sleep health (*χ* ^*2*^(3) = 60.86, *p* < 0.001). The full model was statistically significant (*χ* ^*2*^(6) = 211.59, *p* < 0.001), and classification accuracy was acceptable (66.8%). The fit indices of the model showed a Cox & Shell *R* ^*2*^ of 0.04, and a Nagelkerke *R* ^*2*^ of 0.05, indicating that the model explained a small yet meaningful portion of the variance in sleep health (4%–5%). These findings indicate that both structural and functional aspects of social connectedness, as well as subjective loneliness, are relevant predictors of sleep health.

**TABLE 3:** Results of hierarchical logistic regression models.

Predictor	B	*p*	OR [CI]	Predicted difference (%) [CI]
Model 1				
Functional support	−0.05	< 0.001**	0.95 [0.93, 0.97]	−1.1 [−1.5, −0.7]
Family network	−0.01	0.508	0.99 [0.97, 1.02]	−0.2 [−0.7, 0.3]
Friend network	−0.05	< 0.001**	0.95 [0.93, 0.97]	−1.1 [−1.5, −0.07]
Sometimes lonely	0.59	< 0.001**	1.80 [1.52, 2.13]	13.6 [9.6, 17.7]
Often lonely	0.75	< 0.001**	2.11 [1.50, 2.96]	17.5 [9.2, 25.9]
Mostly lonely	0.62	0.047*	1.86 [1.01, 3.43]	14.4 [−0.5, 29.4]
Model 2				
Functional support	−0.06	< 0.001**	0.95 [0.93, 0.97]	−1.2 [−1.6, −0.8]
Family network	−0.01	0.551	0.99 [0.97, 1.02]	−0.2 [−0.6, 0.3]
Friend network	−0.04	< 0.001**	0.96 [0.94, 0.98]	−0.9 [−1.3, −0.4]
Sometimes lonely	0.51	< 0.001**	1.67 [1.41, 1.98]	11.6 [7.6, 15.6]
Often lonely	0.67	< 0.001**	1.96 [1.39, 2.76]	15.4 [7.2, 23.6]
Mostly lonely	0.50	0.114	1.65 [0.89, 3.05]	11.2 [−3.3, 25.8]
Female	0.50	< 0.001**	1.65 [1.47, 1.86]	10.8 [8.3, 13.3]
Age	0.01	0.063	1.01 [1.00, 1.01]	0.1 [−0.0, 0.2]
SES	−0.05	< 0.001**	0.95 [0.94, 0.97]	−1.0 [−1.4, −0.6]
Model 3				
Functional support	−0.01	0.214	0.99 [0.97, 1.01]	−0.3 [−0.6, 0.1]
Family network	−0.00	0.841	1.00 [0.97, 1.02]	−0.1 [−0.5, 0.4]
Friend network	−0.03	0.019**	0.98 [0.96, 1.00]	−0.5 [−0.9, −0.1]
Sometimes lonely	−0.03	0.794	0.98 [0.81, 1.18]	−0.5 [−4.1, 3.2]
Often lonely	−0.41	0.040*	0.66 [0.45, 0.98]	−7.4 [−14.0, −0.8]
Mostly lonely	−0.28	0.476	0.75 [0.35, 1.64]	−5.2 [−18.9, 8.5]
Female	0.43	< 0.001**	1.54 [1.36, 1.74]	8.4 [5.9, 10.8]
Age	0.01	< 0.001**	1.01 [1.01, 1.02]	0.2 [0.1, 0.3]
SES	−0.03	0.004**	0.97 [0.95, 0.99]	−0.6 [−1.0, −0.2]
Anxiety	0.18	< 0.001**	1.20 [1.17, 1.24]	3.5 [3.1, 4.0]
Life satisfaction	−0.05	< 0.001**	0.95 [0.94, 0.97]	−0.9 [−1.2, −0.7]
Depressive symptoms	0.04	< 0.001**	1.04 [1.03, 1.06]	0.8 [0.5, 1.1]

*Note:* Prediction of poor sleep using binary logistic regressions. Model 1 included only social support predictors. Model 2 additionally controlled for age, SES, and sex, whilst model 3 additionally included anxiety symptoms and life satisfaction. The reference category for the categorical predictor *loneliness* was *never lonely* (= 0). Predicted differences (average marginal effects) are depicted in %, as well as their 95% confidence intervals.

Abbreviation: CI = 95% Confidence Interval.

* *p* < 0.05.

** *p* < 0.001.

### 3.3 Results of the Hierarchical Logistic Regression Model With Covariates

When including age, sex, and SES as covariates (see Table 3, model 2), friend network size, functional social support, and loneliness remained significant predictors of sleep health, whereas family network size did not. Compared to individuals who reported *never feeling lonely*, 11.6% more people experienced poor sleep when they reported feeling *sometimes lonely*, and 15.4% when feeling *often lonely*. Even though the category *mostly lonely* was not significant (*p* = 0.114), the overall association of loneliness with sleep health was (*χ* ^*2*^(3) = 45.19, *p* < 0.001). In comparison to men, 10.8% more women reported poor sleep, and with every one‐point increase in SES, 1.0% fewer people reported poor sleep (all: *p* < 0.001). The association with age (with every additional year, 0.1% more people reported poor sleep) was not significant (*p* = 0.063). The model remained statistically significant (*χ* ^*2*^(9) = 316.44, *p* < 0.001), (Cox & Shell *R* ^*2*^ = 0.06, Nagelkerke *R* ^*2*^ = 0.08) reflecting again a very small portion of explained variance (6%–8%), with an almost unchanged overall classification accuracy (66.7%).

In a third significant model (*χ* ^*2*^(12) = 831.04, *p* < 0.001), adding anxiety, life satisfaction, and depressive symptoms (please see Table 3, model 3), out of the three main predictors, only friend support (*p* = 0.019) remained a significant predictor of sleep health. In contrast, functional social support (*p* = 0.214) and loneliness (*p =* 0.205) were no longer significant.

Again, sex, age (both: *p* < 0.001), and SES (*p* = 0.004) were significant in model 3. With every additional year of age, 0.2% more people reported poor sleep. The three psychological variables were significant predictors (all: *p* < 0.001), too. With each one‐point increase in anxiety symptoms, 3.5% more; with each one‐point increase in depressive symptoms, 0.8% more; and with each one‐point increase in life satisfaction, 0.9% fewer people reported poor sleep. The third model explained notably more variance (14%–20%; Cox & Shell *R* ^*2*^ = 0.14, Nagelkerke *R* ^*2*^ = 0.20), while the overall classification accuracy of the model improved slightly (70.5%).

### 3.4 Subgroup Analysis

We conducted subgroup analyses to examine potential differences in sleep‐related patterns across both sexes (Tables A1 and A2 in the Appendix A) and SES groups (Tables A3 and A4 in the Appendix A). We report the findings from the fully adjusted model and highlight notable associations observed in the model, including sociodemographic covariates. Given their exploratory nature and the multiple comparisons involved, results should be interpreted with caution rather than as confirmatory evidence.

#### 3.4.1 Association of Sleep Health and Social Network Variables Stratified by Sex

In both subgroups, models with sociodemographic covariates (women: *χ* ^*2*^(8) = 145.26, *p* < 0.001; men: *χ* ^*2*^(8) = 120.27, *p* < 0.001) and full models with psychological covariates were significant (women: *χ* ^*2*^(11) = 445.24, *p* < 0.001; men: *χ* ^*2*^(11) = 337.45, *p* < 0.001). In the sociodemographic model, a one‐point increase in functional support was associated with 1.4% fewer women and 0.9% fewer men reporting poor sleep (all: *p* < 0.001). Family network size was not significant, whereas friend network (women: *p* = 0.012; men: *p* < 0.001) and SES (women: *p* = 0.002; men: *p* < 0.001) were negatively associated with poor sleep.

These associations only persisted in men in the fully adjusted model (SES: *p* = 0.020; friend network: *p* = 0.038). Loneliness was no longer associated with sleep health in women (*χ* ^*2*^(3) = 1.03, *p* = 0.794), but was in men (*χ* ^*2*^(3) = 9.64, *p* = 0.022). In both models, age was positively associated with poor sleep in women (both: *p* < 0.001), but not men (both: *p* > 0.979). The psychological predictors were significant in both women and men (depressive symptoms in men: *p* = 0.034; all others: *p* < 0.001). The results of the subgroup analyses indicated that the friend network was especially relevant in men and that age influenced sleep health only in women.

#### 3.4.2 Association of Sleep Health and Social Network Variables Stratified by SES Group

Across all SES groups, both models were statistically significant and demonstrated an acceptable overall accuracy of < 60%: low SES (sociodemographic: *χ* ^*2*^(8) = 39.19, *p* < 0.001; fully adjusted: *χ* ^*2*^(11) = 134.05, *p* < 0.001), middle SES (sociodemographic: *χ* ^*2*^(8) = 181.81; fully adjusted: *χ* ^*2*^(11) = 477.14, both *p* < 0.001), and high SES (sociodemographic: *χ* ^*2*^(8) = 42.68; fully adjusted: *χ* ^*2*^(11) = 176.76, both *p* < 0.001). Anxiety (all: *p* < 0.001), life satisfaction (low: *p* = 0.024; middle and high: *p* < 0.001), and depressive symptoms (low: *p* = 0.006; middle: *p* < 0.001; high: *p* = 0.003) were significant across all SES groups. Differences between SES groups in the fully adjusted model were small and showed no clear pattern: loneliness was significant in the low SES group (*χ* ^*2*^(3) = 8.05, *p* = 0.045) but not in the middle (*χ* ^*2*^(3) = 0.73, *p* = 0.867) or high SES group (*χ* ^*2*^(3) = 5.43, *p* = 0.143). The friend network (*p* = 0.041) predicted sleep only in the middle SES group. Age showed no effect in the low SES group (*p* = 0.324) but was associated with 0.2% and 0.4% more poor sleepers in the middle (*p* = 0.002) and high SES groups (*p* = 0.003), respectively. Female sex was significant in the middle and high SES groups (both: *p* < 0.001).

## 4 Discussion

In this study, we focused on the associations between loneliness, structural support (family and friend network size), and functional support with sleep health in a community‐based adult sample in Germany. One of the unique contributions of this study is its distinction between different forms of social support, specifically differentiating functional and structural dimensions and evaluating their impact on sleep health.

Our results showed that, when including all covariates (model 3), only the friend network remained a significant predictor of sleep health. In this middle‐aged sample, friends might have served as a more readily available source of support, given that parents might have deceased and children often lived farther away. Without psychological covariates, both functional and structural social support, as well as loneliness, were significantly associated with sleep health (model 1) − a finding consistent with prior findings in the literature (e.g., review by Azizi‐Zeinalhajlou et al. 2022; Seo et al. 2025). In our study, these associations persisted even when controlling for age, sex, and SES (model 2), despite accounting for a very small portion of variance (6%–8%). In line with previous findings (e.g., Gyasi et al. 2022; Deng et al. 2023), we found that loneliness was significantly associated with poor sleep health when controlling for sociodemographic predictors (model 2). For example, Cho et al. (2019) found that older adults with objective social isolation may experience sleep disturbance because they *feel* socially isolated, not just because they are deprived of social networks. This finding, however, did not persist in the fully adjusted model.

With respect to sex‐specific patterns, our results (model 3) indicated that more women reported poor sleep health compared to men. Additional subgroup analysis revealed that friend network size and loneliness seemed to only impact men's sleep health. Previous work by Gallicchio et al. (2007) suggested that low social network involvement was linked to poor self‐rated health in older men, while low perceived social support was associated with poor health in older women − a finding we were not able to replicate for women's sleep health.

We observed a sex‐by‐age interaction in the sex‐stratified analysis: increasing age was unrelated to poor sleep health in men but linked to poorer sleep health in women. This sex‐specific finding can be explained by the decrease in sleep health during the menopausal transition, primarily due to hormonal shifts (e.g., review by Haufe and Leeners 2023): Declining levels of oestrogen and, similarly, falling progesterone levels were associated with various poor sleep health outcomes (e.g., poor sleep efficiency and more sleep disruptions).

Our study results (model 3) supported the literature on the negative association of lower SES with sleep health (e.g., Papadopoulos and Etindele Sosso 2023; Etindele Sosso et al. 2022). Additional subgroup analysis, however, did not reveal a clear pattern: Age was not a significant predictor within the low SES group, suggesting that the effect of age may be less salient under conditions of heightened psychosocial stress. Female sex emerged as a significant predictor of poor sleep in the middle and high SES groups, suggesting that the socioeconomic advantage might not fully buffer women's risk for poor sleep.

Overall, the association of age with sleep health in our study was small: With every additional year, only 0.2% more people reported poor sleep health. Our results can be explained by the age range of our sample, focusing on middle‐aged adults: For example, Milner et al. (2016) showed that older age groups showed less mental health improvements with increased social support than people under 30.

### 4.1 Strengths and Limitations

In sum, our results suggest differentiated associations of structural and functional support, with specific relevance of the friend network in middle‐aged adults, which highlights the need to distinguish these two concepts. Our results further point towards a possible sex‐by‐age interaction. The findings highlight that both demographic factors and interpersonal variables might influence sleep health.

A key limitation of this study is its cross‐sectional design, which does not permit causal inferences regarding the relationships between social support and sleep health. Additionally, the sample consisted predominantly of older adults of good health with relatively high life satisfaction, proficient social networks, and low levels of loneliness, which may not be representative of the broader adult population and might have made it difficult to detect larger effects as well as more pronounced subgroup differences. Participants were recruited from the city of Leipzig, a relatively populous and prosperous city in eastern Germany, potentially limiting the generalizability of the findings to more diverse or rural populations. We excluded participants with severe illness, younger than 40, or using sedatives/anxiolytics to reduce confounding, which might have limited generalizability but increased internal validity. Another limitation is that sex was assessed in its biological meaning using a binary categorisation (male/female), restricting the study's ability to capture the full spectrum of sex‐ and gender‐related influences on sleep health, as non‐binary people were not identifiable. Loneliness was assessed with a single CES‐D item, offering a more limited measure than the multi‐item social support scales. Ultimately, while this study focused on the association of social support and sleep health, the reverse direction warrants equal attention. Recent experimental work suggests that sleep deprivation itself can lead to increased social withdrawal and perceived loneliness, creating a self‐reinforcing cycle of social disconnection (Ben Simon and Walker 2018).

Future research should aim to longitudinally capture the evolving relationship between social networks and sleep health, exploring the unique role of the friend network in older adults, to gain a more comprehensive understanding of how these domains influence one another over time, possibly including pre‐ and post‐pandemic data. The relative contribution of stress pathways remains to be clarified and should be further investigated in future studies across diverse populations and contexts. As has been done in our study, we strongly recommend future research to distinguish the concepts of structural and functional support clearly and to use distinct and adequate means to measure them.

### 4.2 Conclusion and Clinical Relevance

Our study showed that social dimensions of sleep health matter, with loneliness and inadequate social support emerging as potential health risk factors in older adults. With respect to the clinical relevance of our findings, more research is needed to shed light on when, under what circumstances, and how social support variables influence sleep outcomes. After understanding this better, possible interventions aimed at improving sleep health could actively account for social support: incorporating both functional and structural aspects into therapeutic strategies, such as fostering emotionally supportive relationships or enhancing social network engagement, could boost resilience against sleep disturbances. In the future, this might call for a clinically personalised, context‐sensitive approach that integrates social and demographic factors into assessment and treatment planning, in line with the socio‐ecological framework of sleep health.

## Author Contributions

Conceptualization: Eva De Camargo, Christian Sander, Project Administration: Kerstin Wirkner; Funding Acquisition: Kerstin Wirkner, Christoph Engel, Steffi G. Riedel‐Heller and Georg Schomerus; Investigation: Kerstin Wirkner, Andrea E. Zülke, Heide Glaesmer, Andreas Hinz, Christoph Engel, Christian Sander and Steffi G. Riedel‐Heller; Resources: Kerstin Wirkner, Steffi G. Riedel‐Heller and Georg Schomerus; Data Curation, Kerstin Wirkner, Andrea E. Zülke, Heide Glaesmer, Andreas Hinz, Kerstin Wirkner, Christoph Engel, Christian Sander; Formal Analysis: Eva De Camargo and Stephanie Schindler; Validation: Christian Sander; Visualisation: Eva De Camargo; Supervision: Georg Schomerus and Christian Sander; Writing – Original Draft Preparation: Eva De Camargo.; Writing – Review and Editing: Stephanie Schindler and Christian Sander. All authors have read and agreed to the published version of the manuscript.

## Funding

This work was supported by Freistaat Sachsen, 713‐241202, 14505/2470.

## Ethics Statement

The LIFE study was conducted according to the guidelines of the Declaration of Helsinki and approved by the Ethics Committee of the Medical Faculty at the University of Leipzig (registration number: 263/09‐ek, date of approval: 8 November 2010).

## Consent

Informed consent was obtained from all subjects involved in the LIFE Adult study.

## Conflicts of Interest

The authors declare no conflicts of interest.

## Data Availability

Restrictions apply to the availability of these data. Data was obtained from the Leipzig Research Center for Civilization Diseases. All data and samples of LIFE are the property of the University of Leipzig and are subject to the Law for the Protection of Informal Self‐Determination in the Free State of Saxony (Saxon Data Protection Act). Use of data can be requested through the LIFE office (https://life.uni‐leipzig.de/).

## Appendix A

**TABLE A1:** Subgroup analyses stratified by sex, including sociodemographic covariates.

	Predictor	*B*	*p*	OR [CI]	Predicted difference [CI]
Women *n* = 2830	Functional Support	−0.06	< 0.001**	0.94 [0.91, 0.96]	−1.4 [−2.0, −0.8]
	Family Network	0.01	0.471	1.01 [0.98, 1.04]	0.3 [−0.5, 1.0]
	Friend Network	−0.03	0.012**	0.97 [0.94, 0.99]	−0.8 [−1.4, −0.2]
	Sometimes lonely	0.47	< 0.001**	1.60 [1.28, 1.98]	11.1 [5.8, 16.3]
	Often lonely	1.01	< 0.001**	2.73 [1.73, 4.32]	23.9 [13.3, 34.5]
	Mostly lonely	0.63	0.093	1.88 [0.90, 3.93]	15.0 [−2.8, 32.9]
	Age	0.02	< 0.001**	1.02 [1.01, 1.02]	0.4 [0.2, 0.5]
	SES	−0.04	0.002**	0.96 [0.94, 0.99]	−0.9 [−1.5, −0.4]
Men *n* = 2558	Functional Support	−0.05	< 0.001**	0.95 [0.93, 0.98]	−0.9 [−1.5, −0.4]
	Family Network	−0.03	0.145	0.98 [0.94, 1.01]	−0.5 [−1.1, 0.2]
	Friend Network	−0.05	< 0.001**	0.95 [0.93, 0.98]	−1.0 [−1.5, −0.4]
	Sometimes lonely	0.61	< 0.001**	1.83 [1.39, 2.43]	13.0 [6.6, 19.4]
	Often lonely	0.14	0.633	1.15 [0.65, 2.01]	2.7 [−8.7, 14.2]
	Mostly lonely	0.21	0.715	1.24 [0.39, 3.91]	4.3 [−19.7, 28.4]
	Age	−0.01	0.137	0.99 [0.99, 1.00]	−0.1 [−0.3, 0.0]
	SES	−0.05	< 0.001**	0.95 [0.93, 0.98]	−1.0 [−1.5, −0.5]

*Note:* Subgroup analyses of the logistic regression model predicting poor sleep health were conducted separately for women and men. Sociodemographic covariates were included. The reference category for the categorical predictor *loneliness* is *never lonely* (= 0). Predicted differences (average marginal effects) are depicted in %, as well as their 95% confidence intervals.

Abbreviation: CI = 95% Confidence Interval.

** *p* < 0.001.

**TABLE A2:** Subgroup analyses of the fully adjusted model predicting poor sleep health, stratified by sex.

	Predictor	*B*	*p*	OR [CI]	Predicted difference [CI]
Women *n* = 2830	Functional Support	−0.02	0.242	0.98 [0.95, 1.01]	−0.4 [−0.9, 0.2]
	Family Network	0.02	0.320	1.02 [0.98, 1.05]	0.4 [−0.3, 1.0]
	Friend Network	−0.02	0.166	0.98 [0.95, 1.01]	−0.4 [−1.0, 0.2]
	Sometimes lonely	−0.08	0.526	0.93 [0.73, 1.18]	−1.6 [−6.4, 3.3]
	Often lonely	−0.08	0.760	0.92 [0.55, 1.54]	−1.6 [−11.9, 8.7]
	Mostly lonely	−0.39	0.397	0.68 [0.27, 1.68]	−7.7 [−24.5, 9.2]
	Age	0.02	< 0.001**	1.02 [1.01, 1.03]	0.4 [0.3, 0.6]
	SES	−0.02	0.132	0.98 [0.95, 1.01]	−0.4 [−1.0, 0.1]
	Anxiety	0.17	< 0.001**	1.19 [1.15, 1.23]	3.6 [2.9, 4.2]
	Life satisfaction	−0.04	< 0.001**	0.96 [0.94, 0.98]	−0.8 [−1.2, −0.4]
	Depressive symptoms	0.05	< 0.001**	1.05 [1.03, 1.07]	1.1 [0.7, 1.4]
Men *n* = 2558	Functional Support	−0.01	0.476	0.99 [0.96, 1.02]	−0.2 [−0.7, 0.3]
	Family Network	−0.02	0.274	0.98 [0.95, 1.02]	−0.4 [−1.0, 0.3]
	Friend Network	−0.03	0.038*	0.97 [0.94, 1.00]	−0.6 [−1.1, −0.0]
	Sometimes lonely	0.09	0.588	1.09 [0.80, 1.49]	15.4 [−4.1, 7.2]
	Often lonely	−0.96	0.004**	0.38 [0.20, 0.73]	−14.0 [−21.3, −6.6]
	Mostly lonely	0.03	0.969	1.03 [0.26, 4.10]	0.5 [−24.4, 25.3]
	Age	0.00	0.979	1.00 [0.99, 1.01]	−0.0 [−0.2, 0.2]
	SES	−0.04	0.020*	0.97 [0.94, 0.99]	−0.6 [−1.1, −0.1]
	Anxiety	0.20	< 0.001**	1.22 [1.17, 1.27]	3.6 [2.9, 4.3]
	Life satisfaction	−0.06	< 0.001**	0.95 [0.93, 0.97]	−1.0 [−1.3, −0.6]
	Depressive symptoms	0.03	0.034*	1.03 [1.00, 1.06]	0.5 [0.0, 0.9]

*Note:* Subgroup analyses of the logistic regression model predicting poor sleep health were conducted separately for women and men. Psychological covariates were included. The reference category for the categorical predictor *loneliness* is *never lonely* (= 0). Predicted differences (average marginal effects) are depicted in %, as well as their 95% confidence intervals.

Abbreviation: CI = 95% Confidence Interval.

* *p* < 0.05.

** *p* < 0.001.

**TABLE A3:** Subgroup analyses stratified by socioeconomic status group, including sociodemographic covariates.

	Predictor	*B*	*p*	OR [CI]	Predicted difference [CI]
Low SES, *n* = 729	Functional Support	−0.05	0.010**	0.95 [0.91, 0.99]	−1.3 [−2.2, −0.3]
	Family Network	−0.03	0.338	0.98 [0.93, 1.03]	−0.6 [−1.8, 0.6]
	Friend Network	−0.04	0.076	0.96 [0.91, 1.01]	−1.0 [−2.1, 0.1]
	Sometimes lonely	0.50	0.016**	1.61 [1.09, 2.38]	11.4 [2.1, 20.8]
	Often lonely	0.13	0.743	1.13 [0.54, 2.40]	3.0 [−15.0, 20.9]
	Mostly lonely	0.07	0.915	1.07 [0.32, 3.58]	1.6 [−27.2, 30.3]
	Age	−0.01	0.079	0.99 [0.97, 1.00]	−0.3 [−0.6, 0.0]
	Female	0.39	0.015**	1.47 [1.08, 2.01]	9.1 [1.8, 16.3]
Middle SES, *n* = 3318	Functional Support	−0.06	< 0.001**	0.95 [0.92, 0.97]	−1.2 [−1.7, −0.7]
	Family Network	−0.01	0.627	0.99 [0.96, 1.02]	−0.2 [−0.8, 0.5]
	Friend Network	−0.04	0.001**	0.96 [0.94, 0.98]	−0.9 [−1.4, −0.4]
	Sometimes lonely	0.58	< 0.001**	1.79 [1.44, 2.23]	13.4 [8.2, 18.6]
	Often lonely	0.87	< 0.001**	2.39 [1.53, 3.73]	20.3 [9.5, 31.0]
	Mostly lonely	0.70	0.096	2.01 [0.89, 4.56]	16.1 [−3.7, 35.9]
	Age	0.01	0.047*	1.01 [1.00, 1.01]	0.2 [0.0, 0.3]
	Female	0.55	< 0.001**	1.73 [1.49, 2.01]	11.8 [8.6, 15.0]
High SES, *n* = 1341	Functional Support	−0.07	0.004**	0.94 [0.90, 0.98]	−1.3 [−2.2, −0.4]
	Family Network	0.01	0.710	1.01 [0.96, 1.06]	0.2 [−0.8, 1.2]
	Friend Network	−0.04	0.082	0.96 [0.92, 1.01]	−0.7 [−1.6, 0.1]
	Sometimes lonely	0.30	0.148	1.35 [0.90, 2.02]	6.1 [−2.5, 14.8]
	Often lonely	0.61	0.129	1.83 [0.84, 4.00]	13.1 [−5.2, 31.3]
	Mostly lonely	0.22	0.784	1.24 [0.27, 5.80]	4.3 [−28.0, 36.6]
	Age	0.01	0.029*	1.01 [1.00, 1.03]	0.3 [0.0, 0.5]
	Female	0.51	< 0.001**	1.67 [1.30, 2.14]	10.2 [5.3, 15.0]

*Note:* Subgroup analyses of the logistic regression model predicting poor sleep and including sociocemographic covariates were conducted separately for SES groups. SES categories are defined as follows: low = [3–9.2], middle = [9.3–15.3], high = [15.4–21]. The reference category for loneliness is *never lonely*. Predicted differences (average marginal effects) are depicted in %, as well as their 95% confidence intervals.

Abbreviation: CI = 95% Confidence Interval.

* *p* < 0.05.

** *p* < 0.001.

**TABLE A4:** Subgroup analyses of the fully adjusted model predicting poor sleep health, stratified by socioeconomic status group.

	Predictor	*B*	*p*	OR [CI]	Predicted difference [CI]
Low SES, *n* = 729	Functional Support	−0.03	0.271	0.98 [0.93, 1.02]	−0.5 [−1.4, 0.4]
	Family Network	−0.02	0.576	0.98 [0.93, 1.04]	−0.3 [−1.5, 0.8]
	Friend Network	−0.03	0.316	0.97 [0.93, 1.03]	−0.5 [−1.6, 0.5]
	Sometimes lonely	−0.12	0.586	0.89 [0.57, 1.37]	−2.5 [−11.3, 6.3]
	Often lonely	−1.03	0.023**	0.36 [0.15, 0.87]	−19.0 [−33.0, −4.9]
	Mostly lonely	−1.56	0.036*	0.21 [0.05, 0.91]	−26.7 [−45.4, −8.0]
	Age	−0.01	0.324	0.99 [0.98, 1.01]	−0.2 [−0.5, 0.2]
	Female	0.27	0.111	1.31 [0.94, 1.83]	5.6 [−1.3, 12.5]
	Anxiety	0.22	< 0.001**	1.25 [1.16, 1.34]	4.6 [3.3, 5.9]
	Life satisfaction	−0.04	0.024**	0.96 [0.93, 1.00]	−0.8 [−1.4, −0.1]
	Depressive symptoms	0.06	0.006**	1.06 [1.02, 1.10]	1.1 [0.3, 1.9]
Middle SES, *n* = 3318	Functional Support	−0.01	0.463	0.99 [0.97, 1.02]	−0.2 [−0.7, 0.3]
	Family Network	−0.00	0.870	1.00 [0.97, 1.03]	−0.1 [−0.7, 0.6]
	Friend Network	−0.03	0.041*	0.97 [0.95, 1.00]	−0.5 [−1.0, −0.0]
	Sometimes lonely	0.68	0.584	1.07 [0.84, 1.36]	1.3 [−3.5, 6.2]
	Often lonely	−0.94	0.713	0.91 [0.55, 1.50]	−1.8 [−11.3, 7.7]
	Mostly lonely	0.24	0.641	1.27 [0.46, 3.51]	4.9 [−16.1, 25.8]
	Age	0.01	0.002	1.01 [1.00, 1.02]	0.2 [0.1, 0.4]
	Female	0.48	< 0.001**	1.61 [1.37, 1.89]	9.4 [6.3, 12.5]
	Anxiety	0.18	< 0.001**	1.20 [1.16, 1.24]	3.5 [2.9, 4.1]
	Life satisfaction	−0.04	< 0.001**	0.96 [0.94, 0.98]	−0.8 [−1.2, −0.5]
	Depressive symptoms	0.04	< 0.001**	1.04 [1.02, 1.06]	0.8 [0.4, 1.2]
High SES, *n* = 1341	Functional Support	−0.02	0.420	0.98 [0.93, 1.03]	−0.4 [−1.2, 0.5]
	Family Network	0.01	0.698	1.01 [0.96, 1.07]	0.2 [−0.8, 1.1]
	Friend Network	−0.01	0.568	0.99 [0.94, 1.03]	−0.2 [−1.0, 0.6]
	Sometimes lonely	−0.30	0.190	0.74 [0.47, 1.16]	−5.0 [−12.1, 2.1]
	Often lonely	−1.01	0.034*	0.36 [0.14, 0.93]	−14.4 [−24.7, −4.2]
	Mostly lonely	−0.51	0.612	0.60 [0.08, 4.30]	−8.1 [−36.1, 19.9]
	Age	0.02	0.003**	1.02 [1.01, 1.03]	0.4 [0.1, 0.6]
	Female	0.46	< 0.001**	1.58 [1.21, 2.06]	8.1 [3.4, 12.8]
	Anxiety	0.18	< 0.001**	1.19 [1.13, 1.26]	3.1 [2.1, 4.1]
	Life satisfaction	−0.07	< 0.001**	0.93 [0.91, 0.97]	−1.2 [−1.7, −0.6]
	Depressive symptoms	0.05	0.003**	1.05 [1.02, 1.09]	0.9 [0.3, 1.5]

*Note:* Subgroup analyses of the fully adjusted logistic regression model predicting poor sleep were conducted separately for SES groups. Psychological covariates were added. SES categories are defined as follows: low = [3–9.2], middle = [9.3–15.3], high = [15.4–21]. The reference category for loneliness is *never lonely*. Predicted differences (average marginal effects) are depicted in %, as well as their 95% confidence intervals.

Abbreviation: CI = 95% Confidence Interval.

* *p* < 0.05.

** *p* < 0.001.
